# Three New Polyynes from *Codonopsis pilosula* and Their Activities on Lipid Metabolism

**DOI:** 10.3390/molecules23040887

**Published:** 2018-04-12

**Authors:** Xiao-Yu Hu, Fu-Ying Qin, Xi-Feng Lu, Lan-Sheng Zhang, Yong-Xian Cheng

**Affiliations:** 1 School of Pharmacy and Chemistry, Dali University, Dali 671000, China; hxy19911010@163.com; 2 Guangdong Key Laboratory for Genome Stability & Disease Prevention, School of Pharmaceutical Sciences, School of Medicine, Shenzhen University Health Science Center, Shenzhen 518060, China; qinfuying@mail.kib.ac.cn (F.-Y.Q.); lxfstart@vip.163.com (X.-F.L.); 3 State Key Laboratory of Phytochemistry and Plant Resources in West China, Kunming Institute of Botany, Chinese Academy of Sciences, Kunming 650201, China; 4 School of Pharmacy, Henan University of Chinese Medicine, Zhengzhou 450008, China; 5 College of Pharmacy, Chengdu University of Traditional Chinese Medicine, Chengdu 610075, China

**Keywords:** *Codonopsis pilosula*, choushenpilosulynes A–C, lipid metabolism

## Abstract

Three new polyynes, named choushenpilosulynes A–C (**1**–**3**), were isolated from an 85% aqueous EtOH extract of the roots of *Codonopsis pilosula* cultivated in Xundian County of Yunnan province, China. Their structures, including the absolute configuration of the glucose residue in **1** and **2**, were determined by spectroscopic analysis and gas chromatography (GC). In addition, biological evaluation shows that all the compounds can inhibit the expression of the squalene monooxygenase (SQLE) gene in HepG2 cells, suggesting that these compounds may be involved in lipid metabolism.

## 1. Introduction

**Figure 1:**
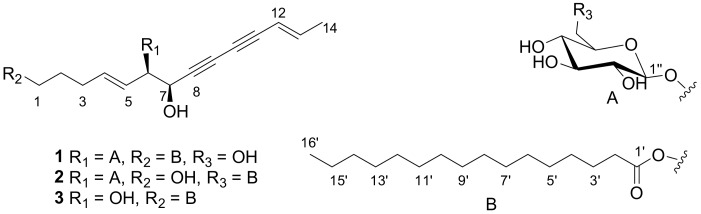
The structures of the compounds choushenpilosulynes A–C (**1**–**3**) from *Codonopsis pilosula*.

High cholesterol is associated with several diseases, including coronary heart disease [1], cancers [2] and neurodegeneration [3]. It has been reported that coronary artery disease (CAD) was the leading cause of death globally and led to over seven million deaths before 2010 [4]. Human 3-hydroxy-3-methylglutaryl-coenzyme A reductase (HMGCR) and squalene monooxygenase (SQLE), two key control enzymes in cholesterol synthesis, play an important role in the metabolism of cholesterol [5]. *Codonopsis pilosula* (Franch.) Nannf. belongs to the family of Campanulaceae and is mainly distributed in northern or western parts of China, such as Gansu and Shaanxi provinces. The dry root of this plant, known as Dangshen, is often utilized in traditional Chinese medicine to replenish Qi, invigorate the spleen, and nourish the lungs [6,7,8]. Previous studies have shown that phytosteroids, sesquiterpenes, triterpenes, alkaloids, alkylalcohol glycosides, phenylpropanoid glycosides, polyacetylene glycosides, neolignan, and polysaccharides are present in this plant [9,10]. However, *C. pilosula* produced in Yunnan province, locally known as Choushen, possesses different functions such as generating fats [11]. This difference attracted our attention and we undertook a study on Choushen grown at high altitudes in Yunnan province, which led to the isolation of three new polyynes, choushenpilosulynes A–C (**1**–**3**) (Figure 1). In this paper, we describe the isolation and structural elucidation of compounds **1**–**3**, as well as their biological activities on lipid metabolism.

## 2. Results and Discussion

### 2.1. Elucidation of the Compounds’ Structures

**Figure 2:**
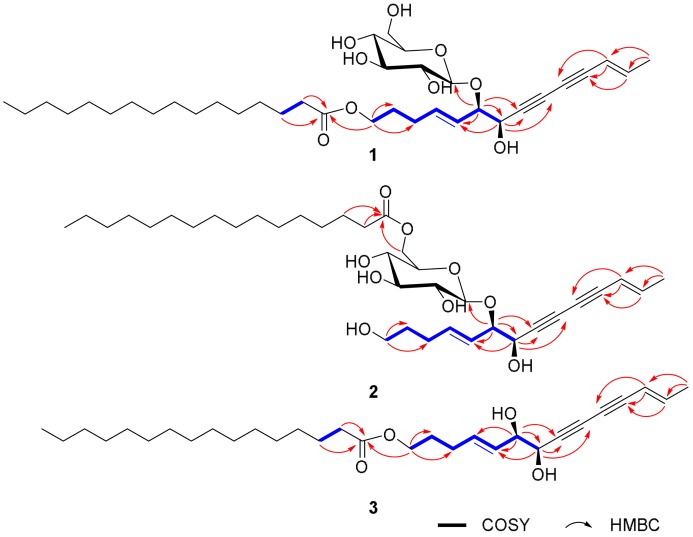
Key COSY and HMBC correlations for **1**–**3**.

Choushenpilosulyne A (**1**), obtained as a white amorphous powder, has a molecular formula C_36_H_58_O_9_ (eight degrees of unsaturation) on the basis of its HRESIMS at *m*/*z* = 657.3985 [M + Na]^+^ (calcd. for 657.3979), ^13^C-NMR, and DEPT spectra (Supplementary Material Figures S5 and S6). The ^1^H-NMR spectrum (Table 1) suggests the existence of two pairs of olefinic protons at *δ*_H_ = 6.31 (1H, dq, *J* = 15.7, 7.0 Hz, H-13), 5.50 (1H, dd, *J* = 15.7, 1.2 Hz, H-12), 5.87 (1H, dt, *J* = 15.6, 6.4 Hz, H-4), and 5.39 (1H, dd, *J* = 15.6, 7.2 Hz, H-5). The large coupling constants (nearly 16.0 Hz) of the two pairs of olefinic protons suggest the *trans* form of the double bonds. The ^13^C-NMR and DEPT spectra (Table 1) show that this substance contains 36 carbons, including two methyl, eighteen methylene (two oxygenated), eleven methine (four sp^2^, seven sp^3^) and five quaternary carbons (including one carboxyl). In addition, the observation of one anomeric proton at *δ*_H_ = 4.37 (d, *J* = 7.2 Hz), five methine (*δ*_C_ = 99.0, 76.2, 75.6, 73.1, and 68.8) and one methylene (*δ*_C_ = 60.2) in the ^1^H- and ^13^C-NMR spectra of **1** reveals the presence of a sugar residue. In the ^13^C-NMR and DEPT spectra, four additional quaternary carbons (*δ*_C_ = 79.1, 77.8, 71.7, and 71.3) are part of one conjugated diyne structure [12,13]. These data are similar to those of lobetyolin [14]. The difference is that **1** contains a 16-carbon side chain, which is absent in lobetyolin. This conclusion is confirmed by the HMBC correlation of H-1/C-1′ (*δ*_C_ = 174.1) (Figure S1). Specifically, the HMBC correlation of H-6/C-1′ (*δ*_C_ = 99.0) indicates the position of the glucose residue. The fragment of C_14_-diendiynetriol is confirmed by the ^1^H–^1^H COSY correlations of H-1/H-2/H-3/H-4/H-5/H-6/H-7 and H-12/H-13/H-14, and by the HMBC correlations of H-6/C-7, C-8, H-7/C-8, C-9, H-12/C-10, C-11, and H-13/C-11 (Figure 2). The relevant NMR data in the literature suggest that *threo* and *erythro vic*-diols with similar partial structures have coupling constants of 6.0–7.0 Hz for *threo* diols and 3.0–4.0 Hz for *erythro* diols [15,16,17,18]. The *J*_H-6,H-7_ (6.5 Hz) value indicates a *threo* configuration between H-6 and H-7. For the configuration of sugar moiety, the presence of a d-glucose residue in the structure of **1** comes from analysis of the acid hydrolysis product. In detail, the l-cysteine methyl ester hydrochloride derivatives of the hydrolysis product of **1**, d- and l-glucose, were prepared and subjected to comparison via gas chromatography (GC) (Supplementary Material Figures S1–S4). The retention time for **1** is 21.246 min, close to that of d-glucose (21.276 min), rather than that of l-glucose (21.768 min), clarifying the type of sugar and its configuration. Taken together, all of these results allow for the identification of the structure of **1**, named choushenpilosulyne A (Supplementary Material Figures S5–S15).

Choushenpilosulyne B (**2**), isolated as a white amorphous powder, has the molecular formula C_36_H_58_O_9_ (8 degrees of unsaturation), deduced from analysis of its HRESIMS *m*/*z* = 657.3973 [M + Na]^+^ (C_36_H_58_NaO_8_, calcd. for 657.3979), ^13^C-NMR, and DEPT spectra (Supplementary Material Figure S16 and S17). The ^1^H-NMR spectrum (Table 2) exhibits two pairs of olefinic protons at *δ*_H_ = 6.32 (1H, dq, *J* = 15.6, 6.7 Hz, H-13), 5.51 (1H, d, *J* = 15.6 Hz, H-12), 5.84 (1H, dt, *J* = 15.5, 6.5 Hz, H-4), and 5.46 (1H, dd, *J* = 15.5, 7.5 Hz, H-5). The ^13^C-NMR and DEPT spectra show four additional quaternary carbons (*δ*_C_ = 79.2, 77.1, 71.8, and 70.8) and four olefinic methine (*δ*_C_ = 144.3, 137.8, 125.2, and 109.5). The data of **2** are very similar to those of **1**, differing in that the side chain in **2** is connected to C-6″, which is verified by the upfield shift of C-1 and downfield shift of glucose C-6 in compound **2** with respect to those in compound **1**, and by the HMBC correlations of Ha-6″ (*δ*_H_ = 4.37)/C-1′ (*δ*_C_ = 174.5). Additionally, the HMBC correlation of H-6/C-7 clearly indicates the location of the sugar moiety. Similarly, the *J*_H-6,H-7_ (6.7 Hz) value indicates a *threo* configuration between H-6 and H-7. A d-glucose residue was identified in the structure of **2** by comparing the retention time of the acid hydrolysis product with the standard sample in the manner as described for **1**. Thus, the structure of **2**, named choushenpilosulyne B, was determined to be that shown in Figure 1 (Supplementary Material Figures S16–S23), Table 2.

Choushenpilosulyne C (**3**), isolated as a white amorphous powder, has the molecular formula C_30_H_48_O_4_ (8 degrees of unsaturation), deduced from analysis of its HRESIMS *m*/*z* = 495.3454 [M + Na]^+^ (C_30_H_48_NaO_4_ calcd. for 495.3450) (Supplementary Material Figures S30–S33), ^13^C-NMR, and DEPT spectra. The ^1^H-NMR spectrum (Table 3) suggests the existence of two pairs of olefinic protons at *δ*_H_ = 6.32 (1H, dq, *J* = 15.7, 6.8 Hz, H-13), 5.57 (1H, overlap, H-12), 5.80 (1H, dt, *J* = 15.5, 6.4 Hz, H-4), and 5.57 (1H, overlap, H-5). In the ^13^C-NMR and DEPT spectra, four additional quaternary carbons (*δ*_C_ = 78.0, 76.5, 72.6, and 71.3) and four olefinic methine (*δ*_C_ = 145.1, 134.2, 130.3, and 110.6) are observed. These data are very similar to those of **1**, differing in that the sugar moiety is absent in **3**. The HMBC correlation of H-1/C-1′ (*δ*_C_ = 175.6) indicates that the side chain is located at C-1. Similarly, the *J*_H-6,H-7_ (6.7 Hz) value indicates a *threo* configuration between H-6 and H-7. In the ROESY spectrum (Figure S29), the correlations of H-3/H-5 and H-12/H-13 suggest the *trans* form of the double bonds. Thus, the structure of **3**, named choushenpilosulyne C, was determined to be that shown in Figure 1 (Supplementary Material Figures S24–S29).

### 2.2. Biological Evaluation

**Figure 3:**
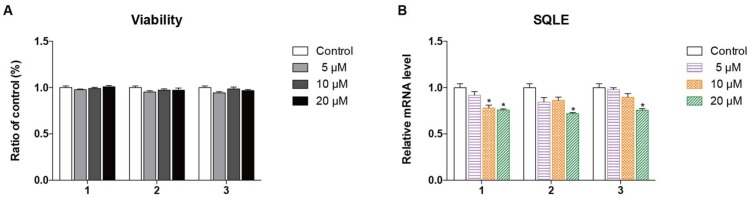
Cytotoxicity of compounds **1**–**3** in HepG2 cells was measured using the MTT assay. Statistical analysis was performed using a one-way analysis of the variance (ANOVA) followed by Bonferroni’s multiple comparison tests. All error bars are S.E.M (**A**). Change in the mRNA expression level of the SQLE gene in cells treated at different concentrations. The transcription level of SQLE gene was normalized by an internal CD36 mRNA control. The data are expressed as the mean ± S.E.M (*n* = 3). * *p* < 0.05 vs. control (**B**).

Squalene monooxygenase (SQLE), as a control enzyme, uses NADPH and molecular oxygen to oxidize squalene to 2,3-oxidosqualene (squalene epoxide). Squalene epoxidase catalyzes the first oxygenation step in sterol biosynthesis and is thought to be one of the rate-limiting enzymes in this pathway. Since SQLE has an important role in lipid biosynthesis and Choushen is known to lower lipids, we studied SQLE. The cytotoxic effects of compounds **1**–**3** on cell viability are shown in Figure 3A. These tests help elucidate the relationship between the compounds and their lipid accumulation inhibitory effects in HepG2 cells. Interestingly, we observed that all the compounds potently reduce the SQLE transcript level in a dose-dependent manner (Figure 3B). Moreover, at the doses tested, none of the compounds affects the viability of hepatic cells, excluding the possibility that their cytotoxicity induces changes in the SQLE transcript level. Therefore, the findings suggest that the isolated compounds might be useful in treating disturbances of the lipid metabolism, such as hypercholesterolemia and atherosclerosis, by regulating cholesterol metabolism. However, the exact molecular mechanism of this regulation needs to be elucidated.

## 3. Experimental Methods

### 3.1. General Procedures

Column chromatography was undertaken on MCI gel CHP 20P (75–150 μm, Mitsubishi Chemical Industries, Tokyo, Japan), Silica gel (200–300 mesh, Qingdao Marine Chemical Inc., Qingdao, China), RP-18 (40–60 µm; Daiso Co., Tokyo, Japan), and Sephadex LH-20 (Amersham Pharmacia, Uppsala, Sweden). Optical rotations were measured on a Horiba SEPA-300 polarimeter (Horiba, Kyoto, Japan). UV spectra were obtained on a Shimadzu UV-2401PC spectrometer (Shimadzu Corporation, Tokyo, Japan). GC analysis was performed using an Agilent 6890N gas chromatography instrument (Agilent Technologies, Santa Clara, CA, USA). Semi-preparative or analytic HPLC was carried out using an Agilent 1200 liquid chromatograph (Agilent Technologies, Santa Clara, CA, USA) where the columns used were a YMC-Pack ODS-A (250 mm × 9.4 mm, i.d., 5 µm), or an Agilent Eclipse XDB-C18 (150 mm × 4.6 mm, i.d., 5 µm). NMR spectra were recorded at room temperature on an AV-400 or AV-600 spectrometer (Bruker, Karlsruhe, Germany) with TMS as an internal standard. ESIMS and HRESIMS data were collected by an Agilent G6230TOF MS spectrometer (Agilent Technologies, Santa Clara, CA, USA).

### 3.2. Plant Material

The roots of *C. pilosula* were collected from a cultivation base in Xundian County, Yunnan province, China, in November 2015. The material was previously identified by Prof. De-Yuan Hong at Beijing Institute of Botany, Chinese Academy of Sciences, China. A voucher specimen (1016268) was deposited at the Herbarium of Kunming Institute of Botany, Chinese Academy of Sciences, Beijing, China.

### 3.3. Extraction and Isolation

The powders of *C. pilosula* (20 kg) roots were soaked with 85% aqueous EtOH (4 × 80 L × 24 h) and concentrated under reduced pressure to yield a crude extract. The extract was suspended in water and partitioned with EtOAc thrice followed by the removal of solvents to produce an EtOAc soluble extract. The EtOAc extract (270.0 g) was separated by using a MCI gel CHP-20 column eluted with gradient aqueous MeOH (35%–100%) to provide six parts (Fr.1–Fr.6). Fr.5 (45.0 g) was further separated by Sephadex LH-20 (MeOH) to yield two fractions (Fr.5.1 and Fr.5.2), of which, the second fraction Fr.5.2 (29.0 g) was divided into three portions (Fr.5.2.1–Fr.5.2.3) by a RP-18 column (MeOH/H_2_O, 35–100%). Among them, Fr.5.2.3 (4.0 g) was purified by Sephadex LH-20 (MeOH), followed by semi-preparative HPLC eluted with MeCN/H_2_O (90%) to yield compound **1** (10.5 mg, R_t_ = 28.2 min), and with MeOH/H_2_O (92%) to yield compound **2** (7.2 mg, R_t_ = 15.8 min). Fr.6 (78.0 g) was separated by Sephadex LH-20 (MeOH) to yield three fractions (Fr.6.1–Fr.6.3). Fr.6.1 (25.0 g) was further separated by a silica gel column (CHCl_3_/MeOH, 50:1, 25:1, 15:1, 9:1, 5:1, 2:1) to get six fractions (Fr.6.1.1–Fr.6.1.6). Of them, Fr.6.1.1 (4.5 g) was purified by Sephadex LH-20 (MeOH) followed by semi-preparative HPLC (MeOH/H_2_O, 92%) to yield compound **3** (5.5 mg, R_t_ = 30.2 min).

### 3.4. Compound Characterization Data

Choushenpilosulyne A (**1**): White amorphous powders; ${\left[\alpha \right]}_{22}^{D}$–14.8 (*c* = 0.3, MeOH); UV (MeOH) *λ*_max_ (log*ε*) = 215 (4.33), 240 (3.80), 254 (3.97), 267 (4.11), 284 (4.02) nm; ESIMS *m*/*z* = 657 [M + Na]^+^, HRESIMS *m*/*z* = 657.3985 [M + Na]^+^ (calcd. for C_36_H_58_NaO_8_, 657.3979); ^1^H- and ^13^C-NMR data, see Table 1.

Choushenpilosulyne B (**2**): White amorphous powders; ${\left[\alpha \right]}_{24}^{D}$–18.0 (*c* = 0.2, MeOH); UV (MeOH) *λ*_max_ (log*ε*) = 215 (4.37), 241 (3.98), 254 (4.08), 268 (4.14), 284 (4.16) nm; ESIMS *m*/*z* = 657 [M + Na]^+^, HRESIMS *m*/*z* = 657.3973 [M + Na]^+^ (calcd. for C_36_H_58_NaO_8_, 657.3979); ^1^H- and ^13^C-NMR data, see Table 2.

Choushenpilosulyne C (**3**): White amorphous powders; ${\left[\alpha \right]}_{24}^{D}$ + 1.6 (*c* = 0.2, MeOH); UV (MeOH) *λ*_max_ (log*ε*) = 215 (4.34), 241 (3.74), 254 (3.98), 267 (4.15), 283 (4.06) nm; ESIMS *m*/*z* = 495 [M + Na]^+^, HRESIMS *m*/*z* = 495.3454 [M + Na]^+^(C_30_H_48_NaO_4_ calcd. for 495.3450); ^1^H- and ^13^C-NMR data, see Table 3.

### 3.5. Acid Hydrolysis and Sugar Analysis

d or l-glucose (1 mg) was dissolved in anhydrous pyridine (1 mL). To these solutions, l-cysteine methyl ester hydrochloride (3.0 mg) was added and the mixtures were stirred at 60 °C for 1 h and concentrated in vacuo at 0 °C. A 0.4 mL solution of 1-(trimethylsiyl) imidazole was slowly added to the mixtures and followed by stirring at 60 °C for 1 h. After cooling, 1 mL of water was slowly added into the mixtures. Then, the mixtures were extracted with *n*-hexane. The *n*-hexane layer was directly analyzed by gas chromatography (GC).

Compound **1** or **2** (3.0 mg, respectively) were stirred at 75 °C with 6 mol/L HCl (1.5 mL) for 5 h. After reaction, the mixtures were extracted with EtOAc (1.5 mL, 3 times). The aqueous layer was neutralized with 1 N NaOH and concentrated in vacuo. Then, the residue was dissolved in anhydrous pyridine (2 mL). To these solutions, l-cysteine methyl ester hydrochloride (3.0 mg) was added. The mixtures were stirred at 60 °C for 1 h and concentrated in vacuo at 0 °C. A 0.4 mL solution of 1-(trimethylsiyl) imidazole was slowly added to the mixtures and followed by stirring at 60 °C for 1 h. After cooling, 1 mL of water was slowly added into the mixtures. Then, the mixtures were extracted with *n*-hexane. The *n*-hexane layer was directly analyzed by gas chromatography (GC). The d-configuration of glucose in **1** or **2** was determined by comparing the retention time with a standard sample [19].

### 3.6. Cell Viability Assay

Cytotoxicity was determined by the MTT assay using previously described method [20]. HepG2 cells (8000 cells/well) were seeded onto a 96-well plate and incubated for 24 h to allow cell adherence. Fresh medium containing the test samples was added into the cultures and incubated at 37 °C for 24 h. MTT was added; after 4 h of incubation, absorbance was measured at a wavelength of 570 nm using an enzyme-linked immunosorbent assay reader (Bio Tek, Winnooski, VT, USA).

### 3.7. RT-PCR

All of RNA in the HepG2 cells was extracted using the Trizol and Direc-zol^TM^ RNA MiniPrep kits. 1 mg total RNA was reversely transcribed with Prime ScriptTM RT Master Mix. SYBR green real-time quantitative assays were performed on a qTOWER apparatus using an SYBR^®^ Premix Ex Taq^TM^ II kit reagent, following the protocol specified by the manufacturer. The expressions of SQLE gene were analyzed. Primers of SQLE used in the study were forward: 5′-cctgaatcagaaaataaggagca-3′, and reverse: 5′-gcttgtttctgaaatattggttcc-3′.

## 4. Conclusions

To conclude, this study led to the isolation of three new polyynes. These compounds were found to inhibit the expression of SQLE gene transcript in HepG2 cells, which suggests that these compounds might contribute to lipid metabolism.

## Figures and Tables

**Table 1:** ^1^H- (600 MHz) and ^13^C-NMR (150 MHz) data of **1** in CDCl_3_ (*δ* in ppm, *J* in Hz).

Position	*δ*_H_	*δ*_C_		*δ*_H_	*δ*_C_
1	4.07, m	63.5 t	5′	1.26, overlap	29.2 ^a^ d
2	1.74, m	27.9 t	6′	1.26, overlap	29.4 ^a^ d
3	2.16, m	28.7 t	7′	1.26, overlap	29.6 ^a^ d
4	5.87, dt (15.6, 6.4)	137.2 d	8′	1.26, overlap	29.7 ^a^ d
5	5.39, dd (15.6, 7.2)	125.4 d	9′	1.26, overlap	29.6 ^a^ d
6	4.22, t (6.5)	81.1 d	10′	1.26, overlap	29.7 ^a^ d
7	4.40, d (6.5)	65.5 d	11′	1.26, overlap	29.7 ^a^ d
8		79.1 s	12′	1.26, overlap	29.7 ^a^ d
9		71.3 s	13′	1.26, overlap	29.7 ^a^ d
10		77.8 s	14′	1.26, overlap	31.8 t
11		71.7 s	15′	1.29, overlap	22.7 t
12	5.50, dd (15.7, 1.2)	109.7 d	16′	0.88, t (6.9)	14.2 q
13	6.31, dq (15.7, 7.0)	144.3 s	1″	4.32, d (7.8) ^b^	99.0 d
14	1.81, d (7.0)	18.9 q	2″	3.47, m	73.1 d
1′		174.1 s	3″	3.23, m	75.6 d
2′	2.29, t (7.6)	34.4 t	4″	3.62, m	68.8 d
3′	1.61, m	25.0 d	5″	3.50, m	76.2 d
4′	1.29, m	29.7 ^a^ t	6″	3.84, m	60.2 t

^a^ These signals can be exchangeable. ^b^ Observed in methanol-*d*_4_.

**Table 2:** ^1^H- (600 MHz) and ^13^C-NMR (150 MHz) data of **2** in CDCl_3_ (*δ* in ppm, *J* in Hz).

Position	*δ*_H_	*δ*_C_		*δ*_H_	*δ*_C_
1	3.63, m	61.6 t	5′	1.26, overlap	29.3 ^a^ d
2	1.69, m	31.2 t	6′	1.26, overlap	29.4 ^a^ d
3	2.20, m	28.7 t	7′	1.26, overlap	29.6 ^a^ d
4	5.84, dt (15.5, 6.5)	137.8 d	8′	1.26, overlap	29.7 ^a^ d
5	5.46, dd (15.5, 7.5)	125.2 d	9′	1.26, overlap	29.8 ^a^ d
6	4.18, t (6.7)	81.7 d	10′	1.26, overlap	29.7 ^a^ d
7	4.42, d (6.7)	65.6 d	11′	1.26, overlap	29.7 ^a^ d
8		79.2 s	12′	1.26, overlap	29.7 ^a^ d
9		70.8 s	13′	1.26, overlap	29.7 ^a^ d
10		77.7 s	14′	1.26, overlap	31.8 t
11		71.8 s	15′	1.30, overlap	22.7 t
12	5.51, d (15.6)	109.5 d	16′	0.88, t (6.9)	14.1 q
13	6.32, dq (15,7, 6.7)	144.3 s	1″	4.32, d (7.8) ^b^	99.3 d
14	1.81, d (6.7)	18.9 q	2″	3.47, m	73.1 d
1′		174.5 s	3″	3.23, m	73.9 d
2′	2.35, t (7.5)	34.2 t	4″	3.62, m	70.4 d
3′	1.62, m	24.9 d	5″	3.50, m	76.1 d
4′	1.31, m	29.4 ^a^ t	6″	Ha 4.37, m Hb 4.23, m	63.5 t

^a^ These signals can be exchangeable. ^b^ *J* was observed in methanol-*d*_4_.

**Table 3:** ^1^H- (400 MHz) and ^13^C-NMR (150 MHz) data of **3** in methanol-*d*_4_ (*δ* in ppm, *J* in Hz).

Position	*δ*_H_	*δ*_C_		*δ*_H_	*δ*_C_
1	4.10, t (6.4)	61.6 t	5′	1.26, overlap	30.8 ^a^ d
2	1..75, m	29.1 t	6′	1.26, overlap	30.8 ^a^ d
3	2.17, m	30.2 t	7′	1.26, overlap	30.8 ^a^ d
4	5.80, dt (15.5, 6.4)	134.2 d	8′	1.26, overlap	30.8 ^a^ d
5	5.57, overlap	130.3 d	2′	2.35, t (7.5)	35.1 t
6	3.99, t (6.7)	81.6 d	3′	1.61, m	26.1 d
7	4.21, d (6.7)	67.7 d	4′	1.29, m	29.4 ^a^ t
8		78.0 s	9′	1.29, overlap	30.8 ^a^ d
9		71.3 s	10′	1.29, overlap	30.7 ^a^ d
10		76.5 s	11′	1.29, overlap	30.6 ^a^ d
11		72.6 s	12′	1.29, overlap	30.5 ^a^ d
12	5.57, overlap	110.6 d	13′	1.29, overlap	30.4 ^a^ d
13	6.32, dq (15.7, 6.8)	145.1 s	14′	1.29, overlap	33.1 t
14	1.81, d (6.1)	18.9 q	15′	1.29, overlap	23.8 t
1′		175.6 s	16′	0.90, t (6.9)	14.5 q

^a^ These signals can be exchangeable.

## Supplementary Materials

The following are available online. Figures S1–S4: GC analysis of the derivative of d-glucose or l-glucose, Figures S5–S13: NMR spectra of **1**, Figures S14 and S15: ESIMS and HRESIMS of **1**, Figures S16–S21: NMR spectra of **2**, Figures S22 and S23: ESIMS and HRESIMS of **2**, Figures S24–S29: NMR spectra of **3**, Figures S30 and S31: ESIMS and HRESIMS of **3**.
