# Supplementary material for: Three New Polyynes from Codonopsis pilosula and Their Activities on Lipid Metabolism
Source: Molecules. 2018 Apr 12;23(4):887. doi: 10.3390/molecules23040887 (PMC6017398; doi:10.3390/molecules23040887)
Supplement: Supplementary file 1 [file molecules-23-00887-s001.pdf]

# Supplementary Materials: Three New Polyynes from *Codonopsis pilosula* and Their Activities on Lipid Metabolism

Xiao-Yu Hu, Fu-Ying Qin, Xi-Feng Lu, Lan-Sheng Zhang, and Yong-Xian Cheng

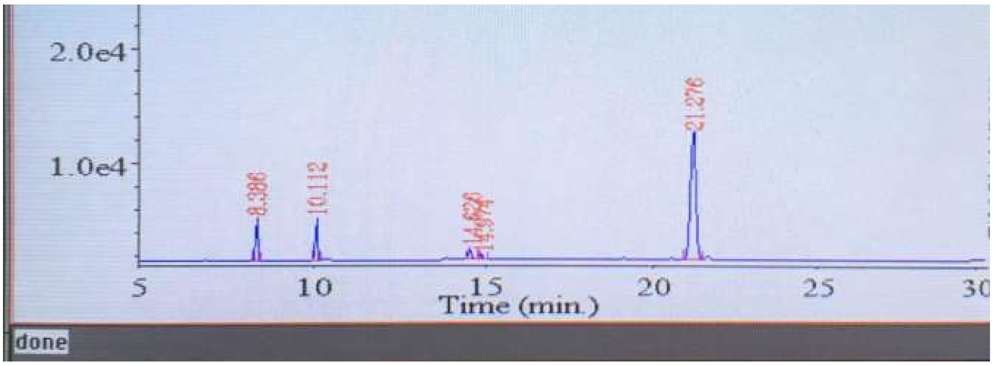


Figure S1. GC analysis of the derivative of D-glucose

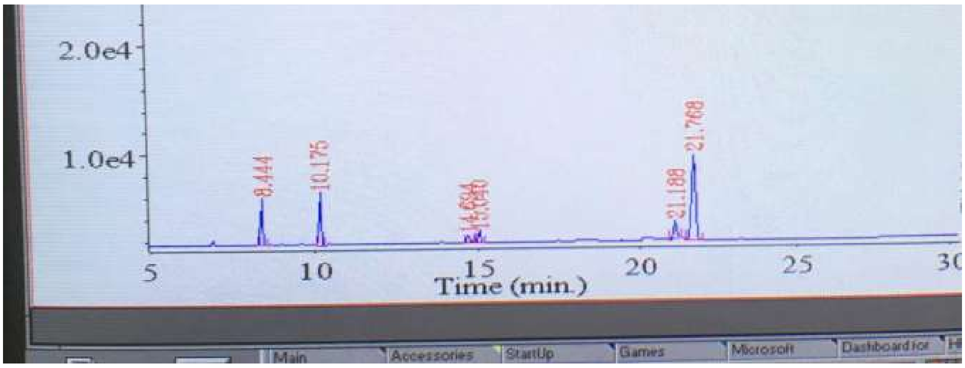


Figure S2. GC analysis of the derivative of L-glucose

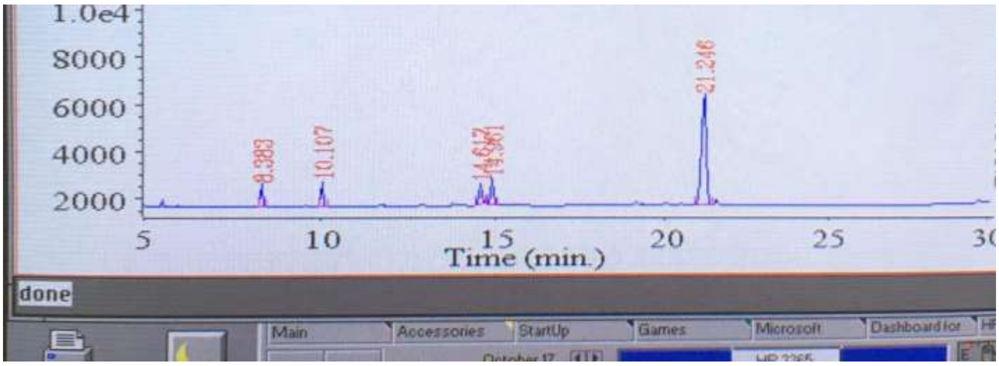


Figure S3. GC analysis of the derivative of compound **1** after hydrolysis

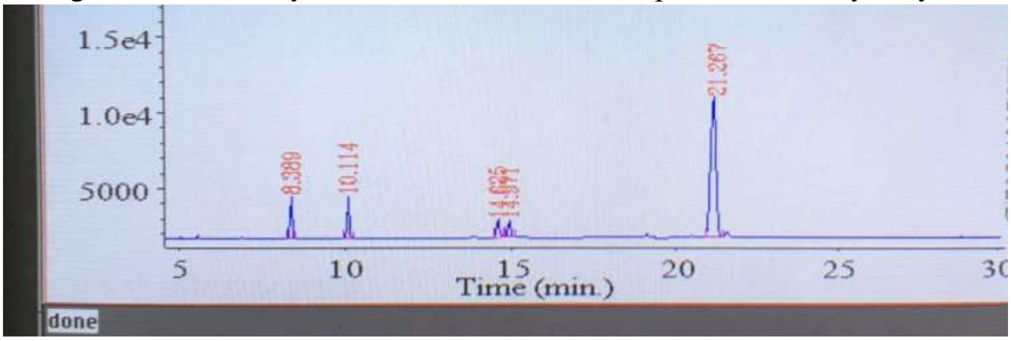


Figure S4. GC analysis of the derivative of compound **2** after hydrolysis

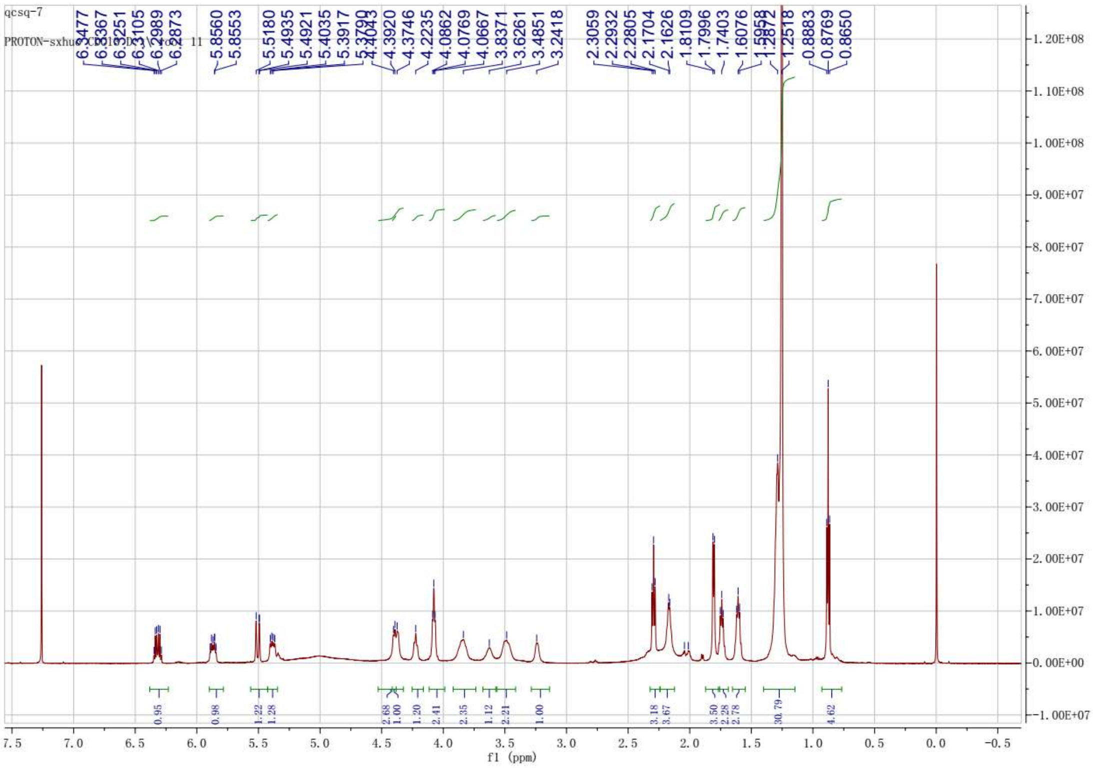


Figure S5.$^{1}$H NMR spectrum of **1** in $CDCl_3$

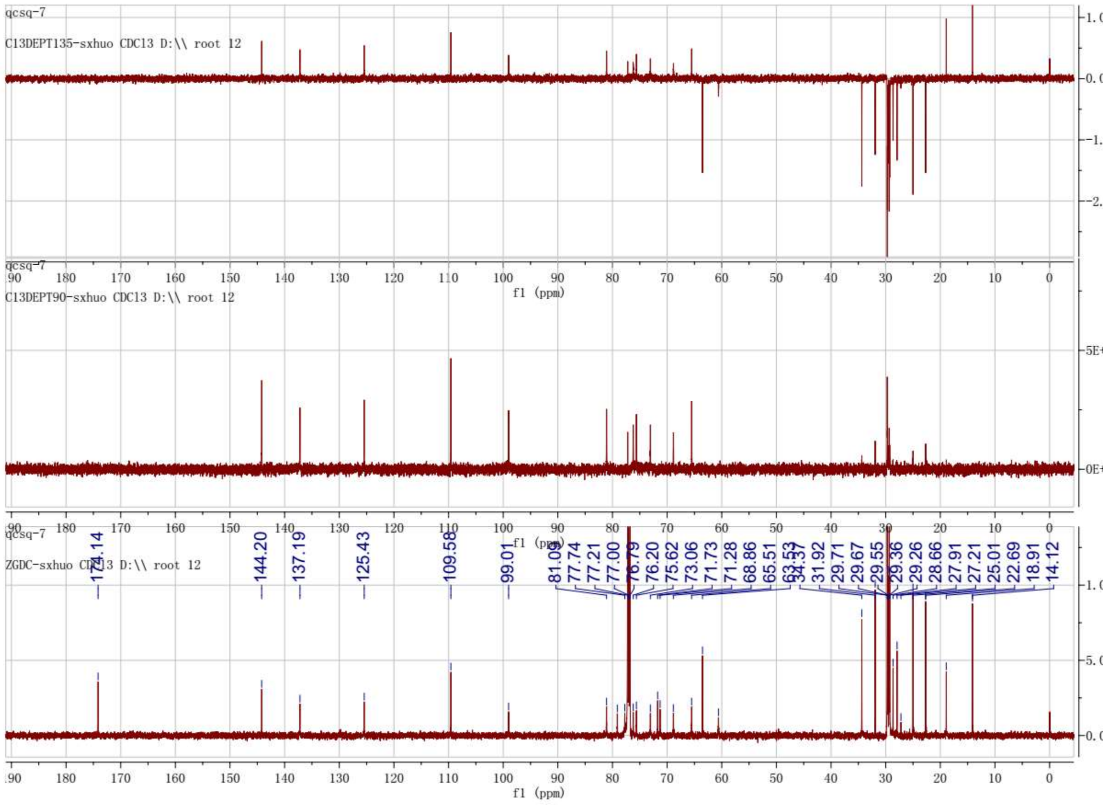


Figure S6. $^{13}$C NMR and DEPT spectra of **1** in $CDCl_3$

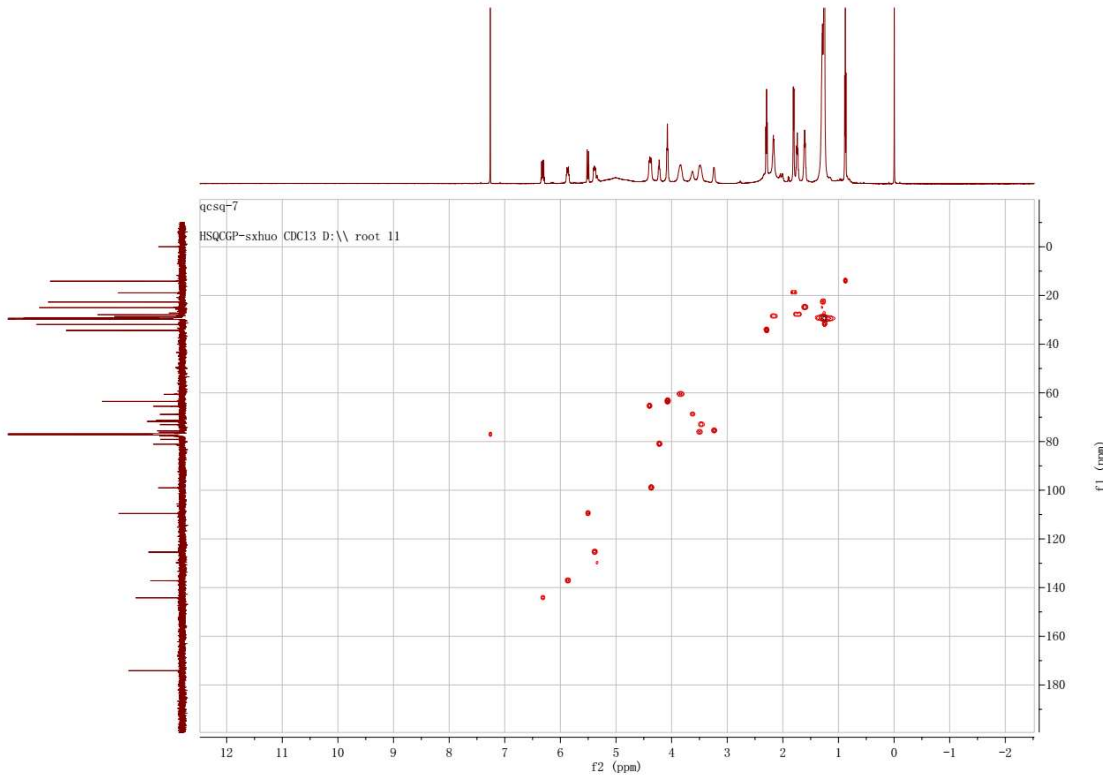


Figure S7. HSQC spectrum of **1** in $CDCl_3$

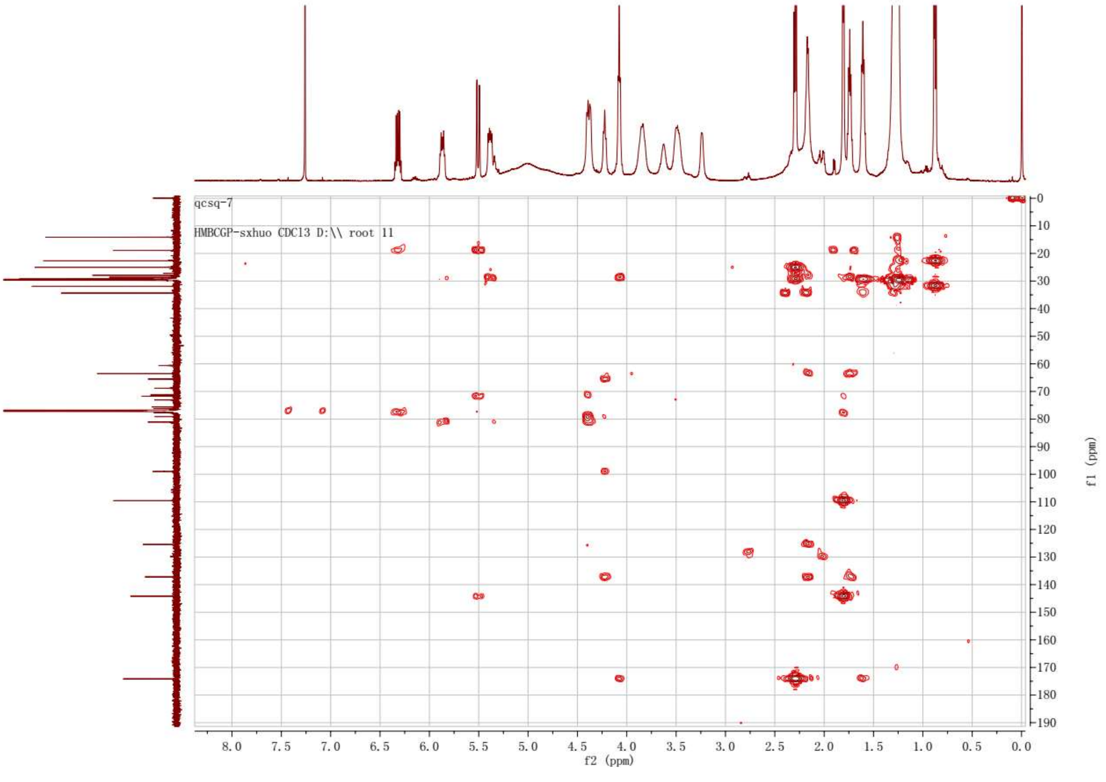


Figure S8. HMBC spectrum of **1** in $CDCl_3$

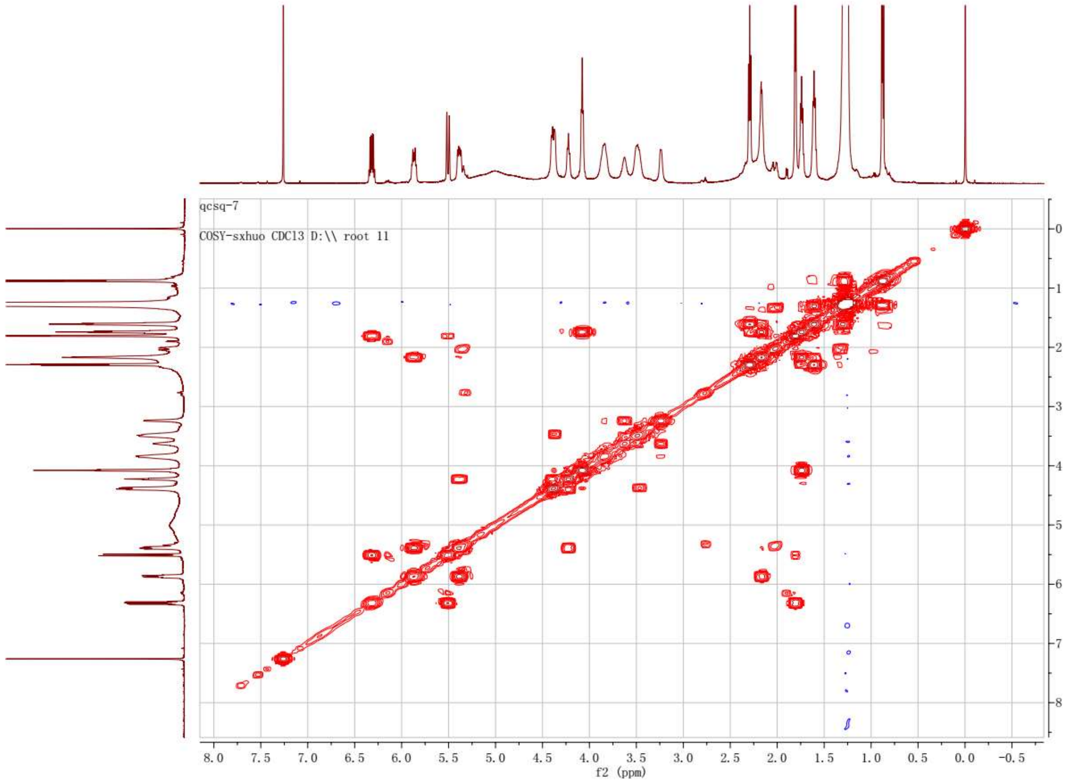


Figure S9. $^{1}H$-$^{1}H$ COSY spectrum of **1** in $CDCl_3$

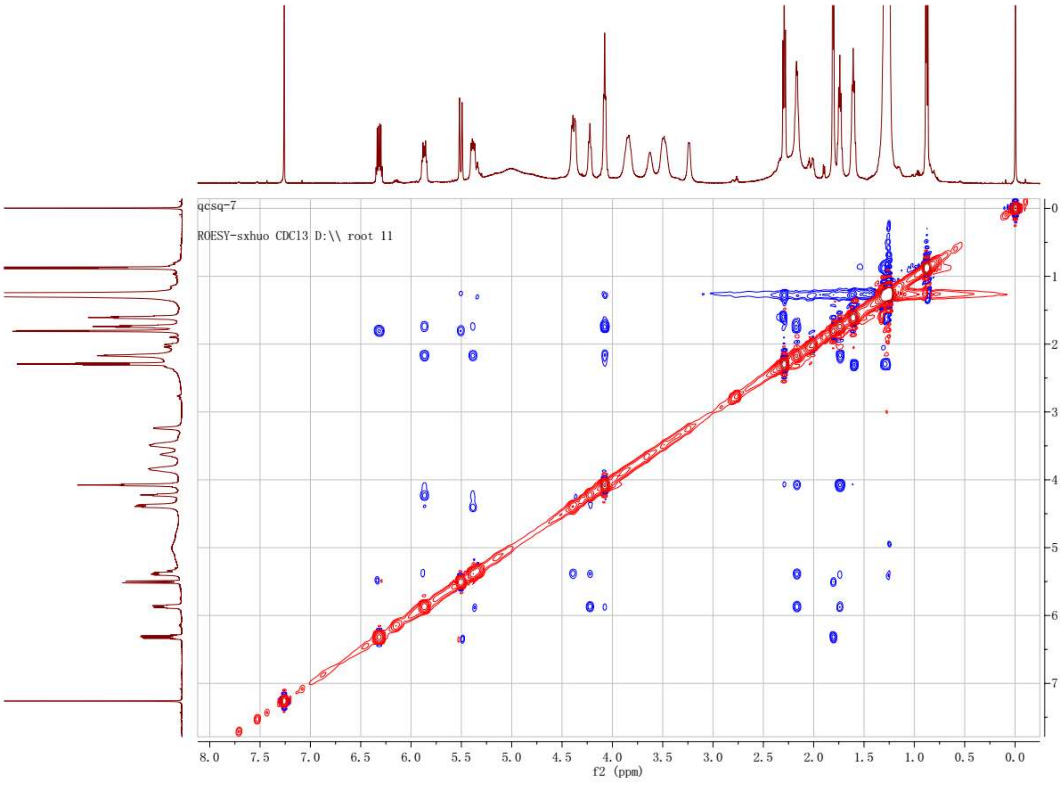


Figure S10. ROESY spectrum of **1** in $CDCl_3$

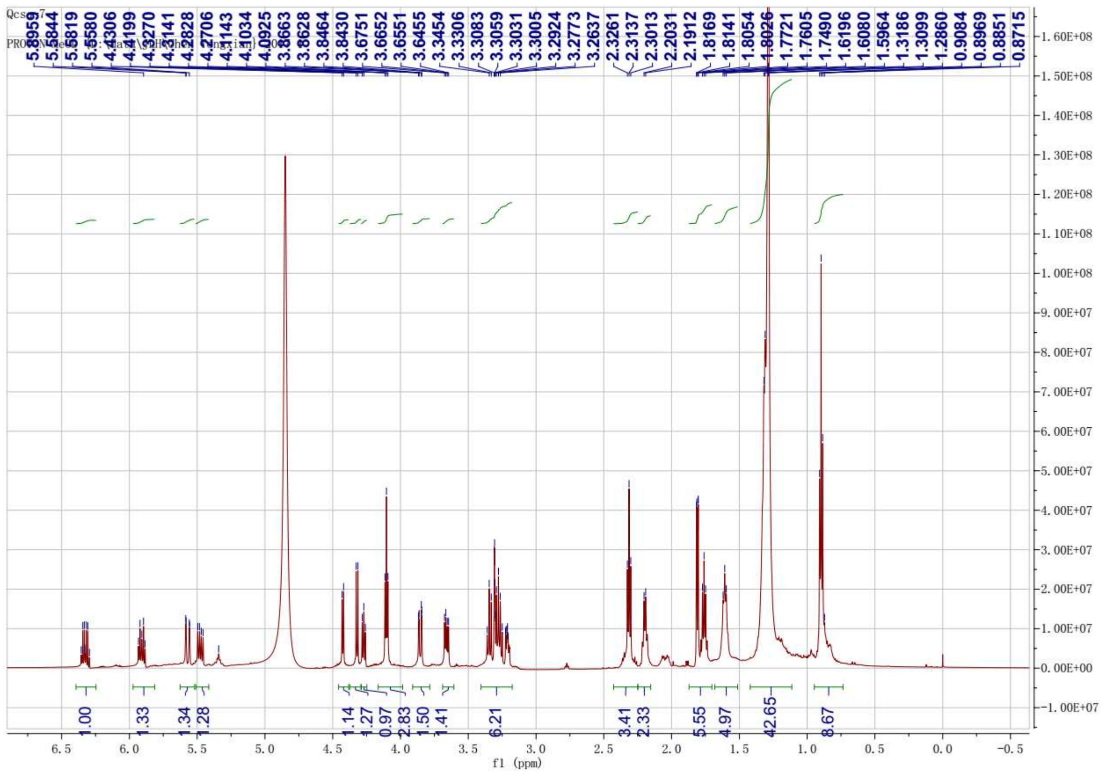


Figure S11.$^{1}$H NMR spectrum of **1** in Methanol-$d_4$

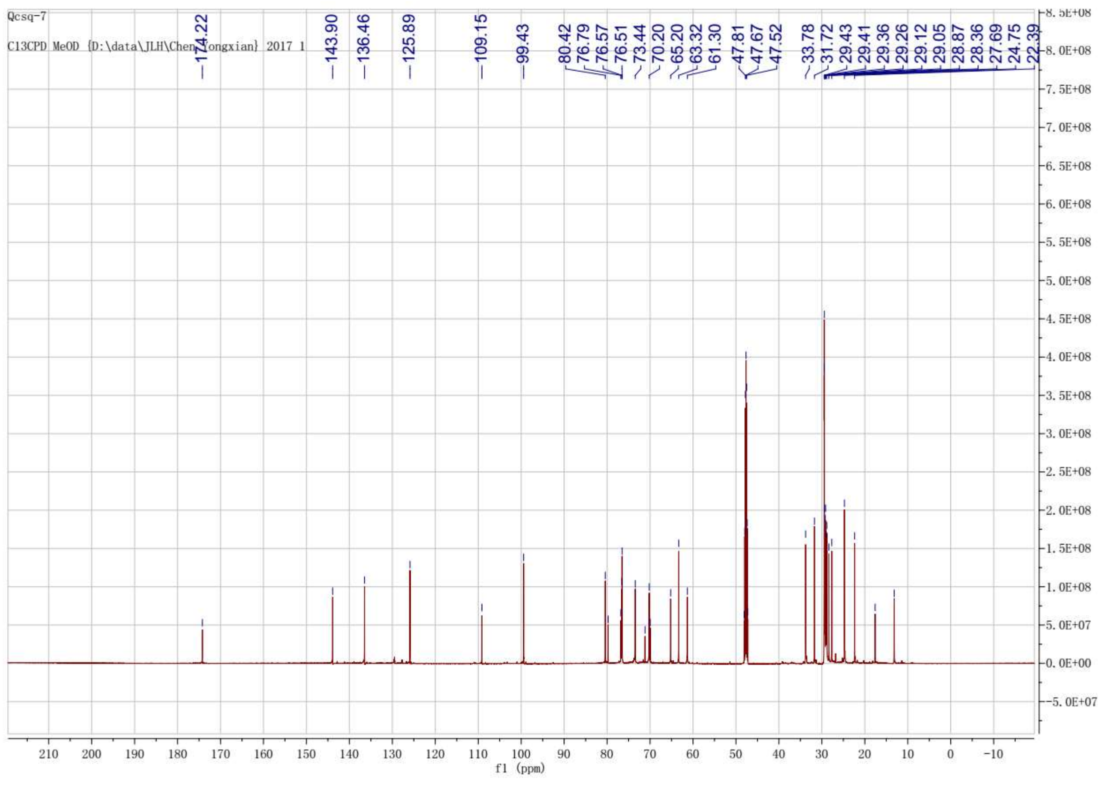


Figure S12.$^{13}$C NMR spectrum of **1** in Methanol-$d_4$

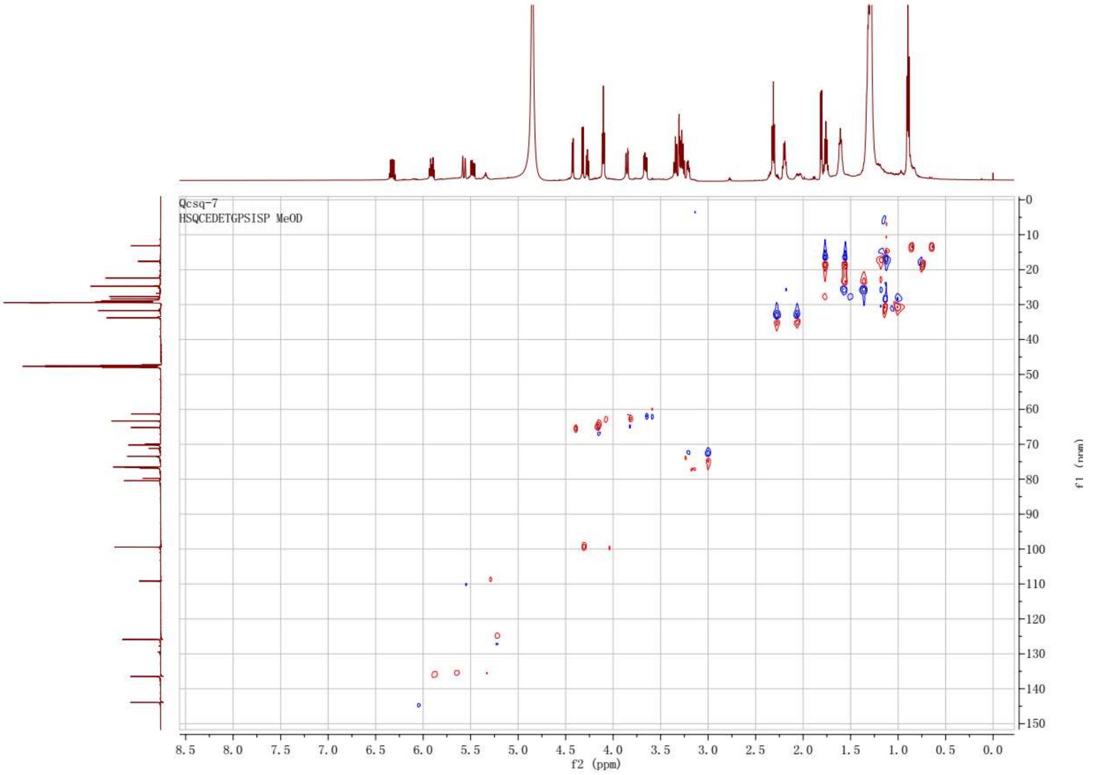


Figure S13. HSQC spectrum of **1** in Methanol-$d_4$

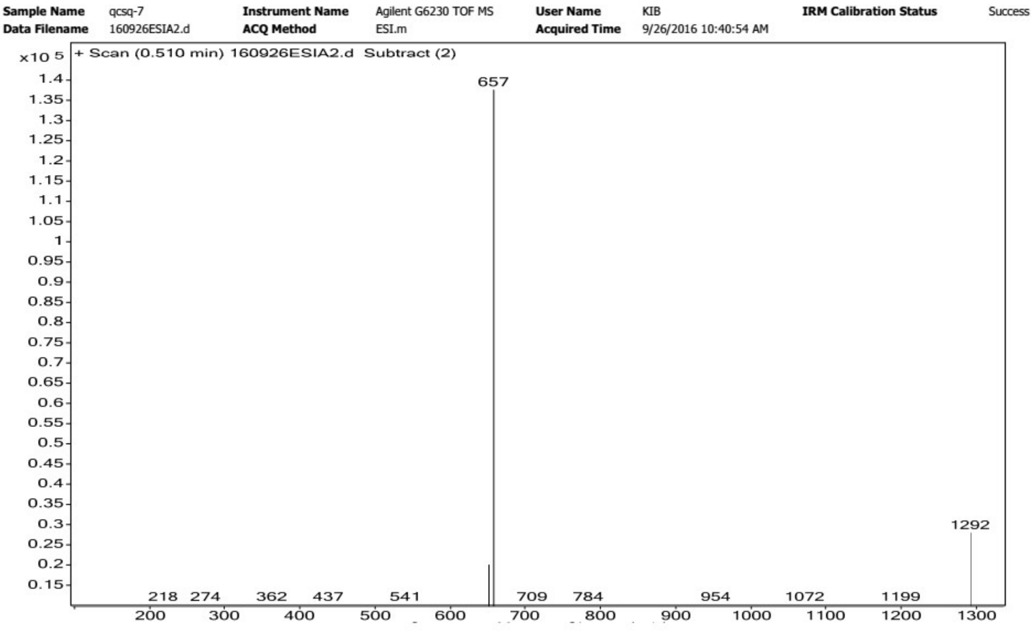


Figure S14. ESIMS of **1**

# Qualitative Analysis Report

| | | | |
|---|---|---|---|
| **Data Filename** | 160926ESIA2.d | **Sample Name** | qcsq-7 |
| **Sample Type** | Sample | **Position** | |
| **Instrument Name** | Agilent G6230 TOF MS | **User Name** | KIB |
| **Acq Method** | ESI.m | **Acquired Time** | 9/26/2016 10:40:54 AM |
| **IRM Calibration Status** | Success | **DA Method** | Default.m |
| **Comment** | | | |

| | | |
|---|---|---|
| **Sample Group** | | **Info.** |
| **Acquisition SW Version** | 6200 series TOF/6500 series Q-TOF B.05.01 (B5125.2) | |

## User Spectra

| Fragmentor Voltage | Collision Energy | Ionization Mode |
|---|---|---|
| 200 | 0 | ESI |

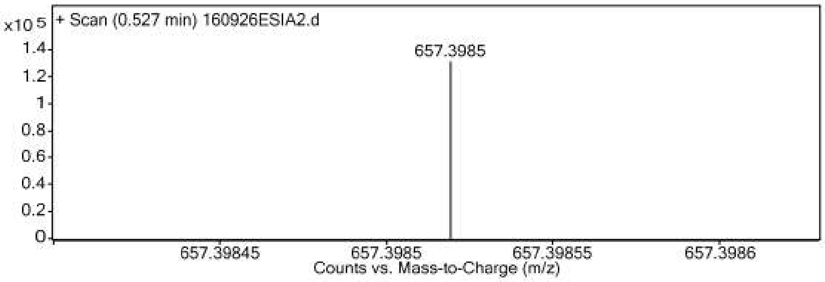


**Peak List**

| *m/z* | z | Abund | Formula | Ion |
|---|---|---|---|---|
| 361.1688 | 1 | 73661.73 | | |
| 362.1697 | 1 | 19172.02 | | |
| 563.2517 | 1 | 144029.48 | | |
| 564.2528 | 1 | 60021.64 | | |
| 565.2505 | 1 | 30172.1 | | |
| 652.4424 | 1 | 21541.73 | | |
| 657.3985 | 1 | 130671.76 | C36 H58 Na O9 | M+ |
| 658.4017 | 1 | 41275.9 | C36 H58 Na O9 | M+ |
| 1291.805 | 1 | 28297.47 | | |
| 1292.8084 | 1 | 21611.54 | | |

**Formula Calculator Element Limits**

| Element | Min | Max |
|---|---|---|
| C | 0 | 200 |
| H | 0 | 400 |
| O | 0 | 12 |
| Na | 1 | 1 |

**Formula Calculator Results**

| Formula | CalculatedMass | Mz | Diff.(mDa) | Diff. (ppm) | DBE |
|---|---|---|---|---|---|
| C36 H58 Na O9 | 657.3979 | 657.3985 | -0.7 | 1.0 | 7.5 |

--- End Of Report ---

Figure S15. HRESIMS of **1**

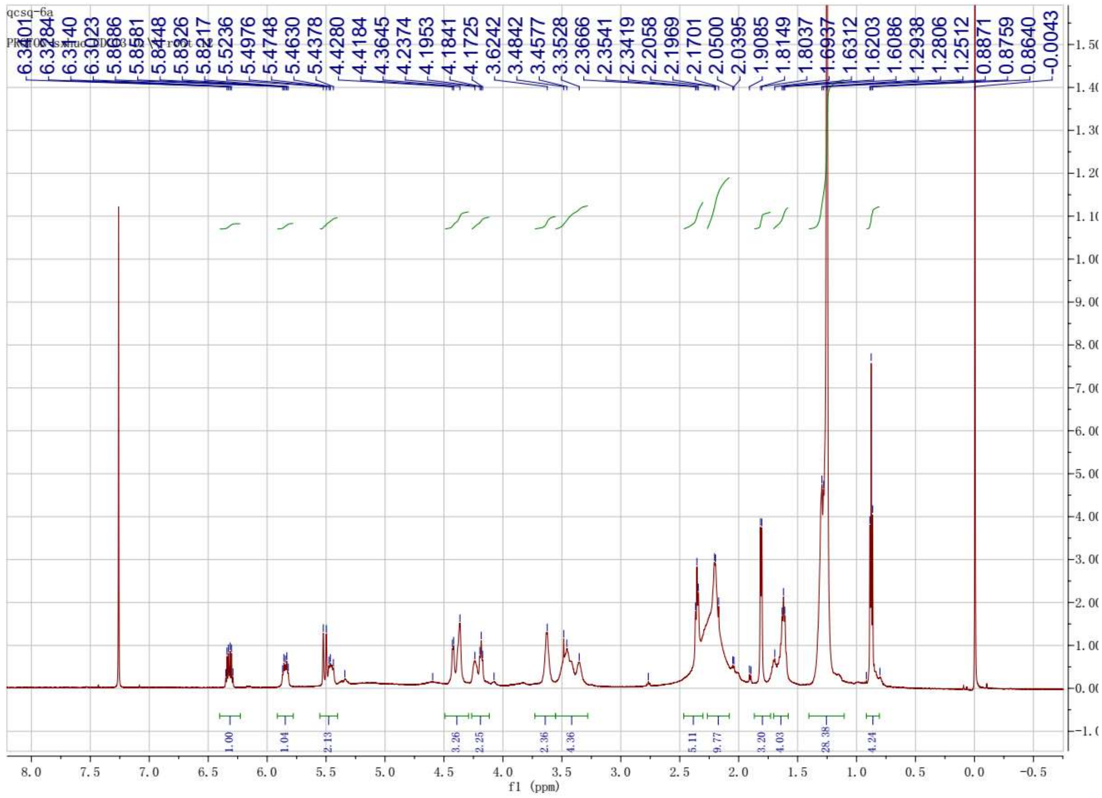

Figure S16. $^{1}$H NMR spectrum of **2** in $CDCl_3$

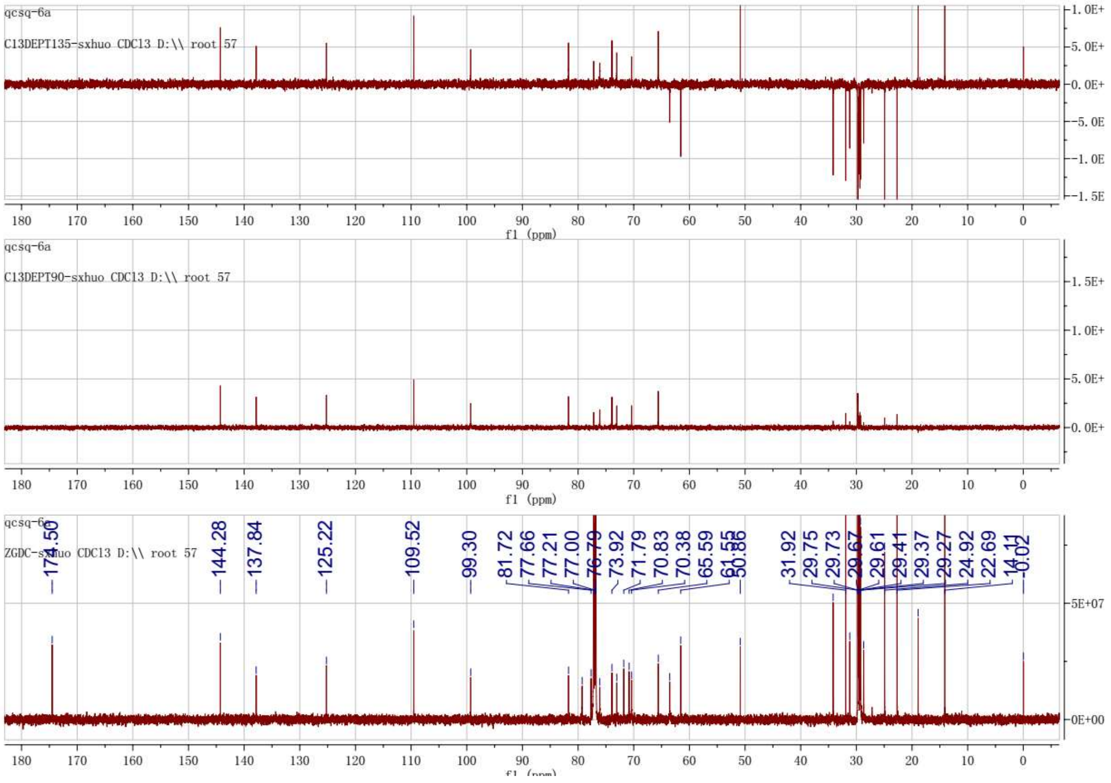

Figure S17. $^{13}$C NMR and DEPT spectra of **2** in $CDCl_3$

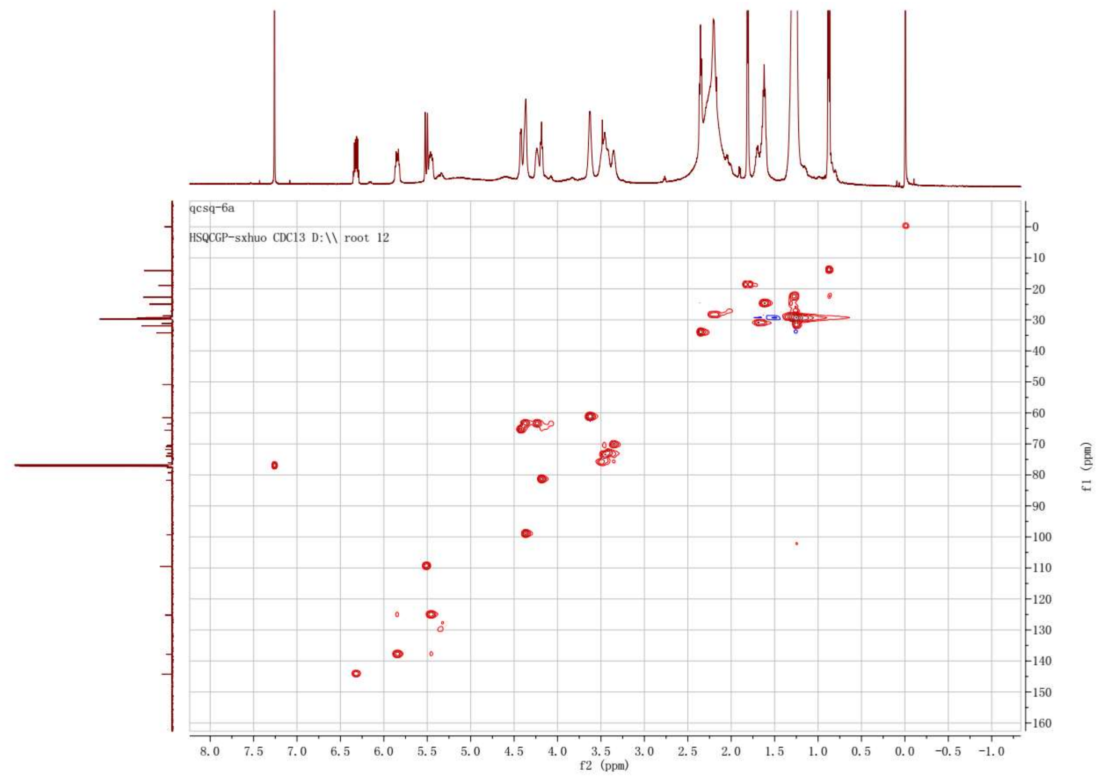


Figure S18. HSQC spectrum of **2** in $CDCl_3$

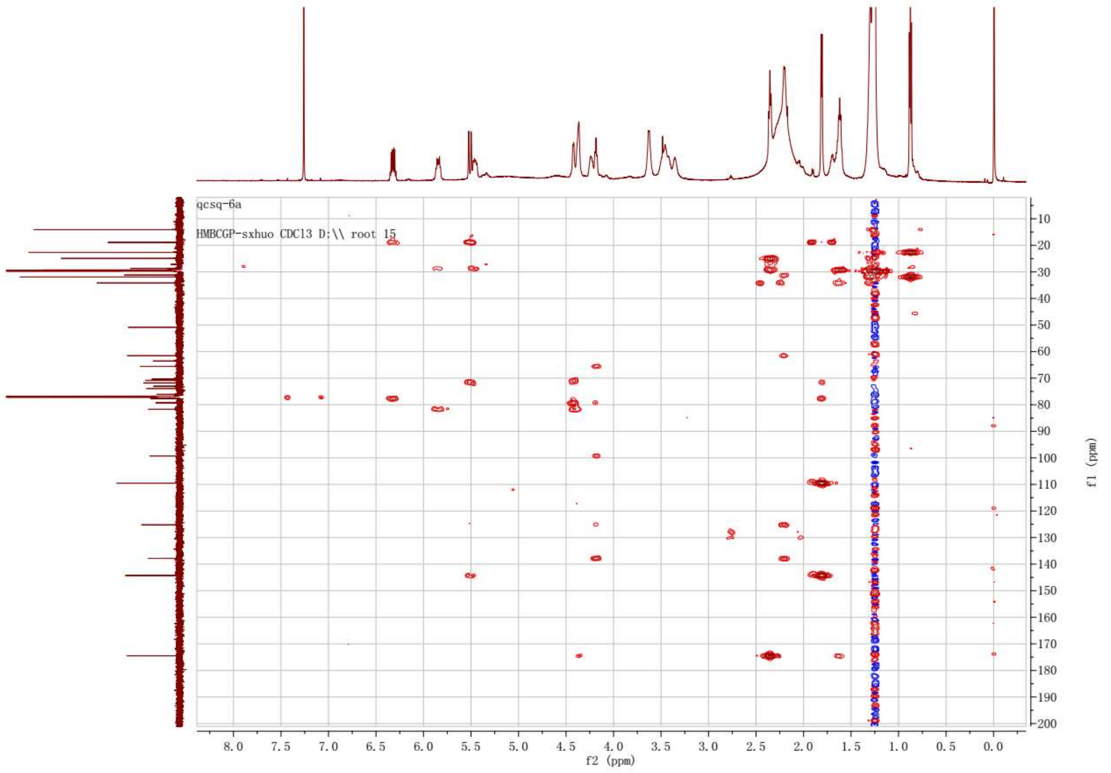


Figure S19. HMBC spectrum of **2** in $CDCl_3$

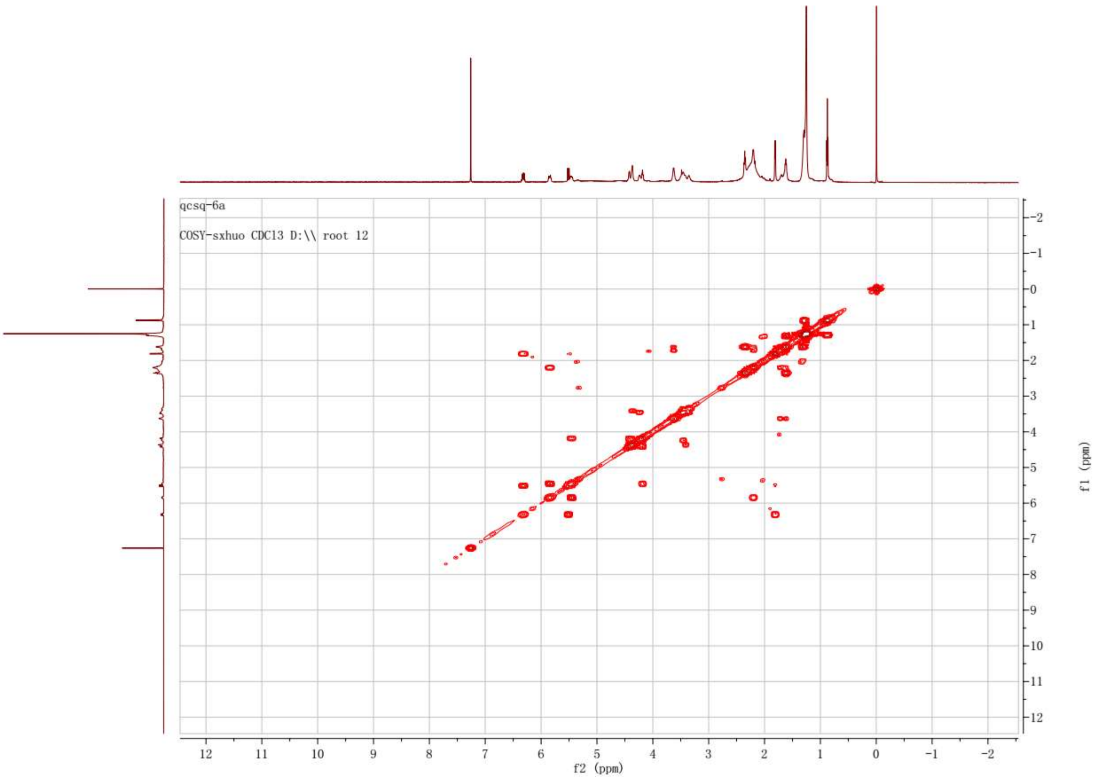

Figure S20. $^{1}$H-$^{1}$H COSY spectrum of **2** in $CDCl_3$

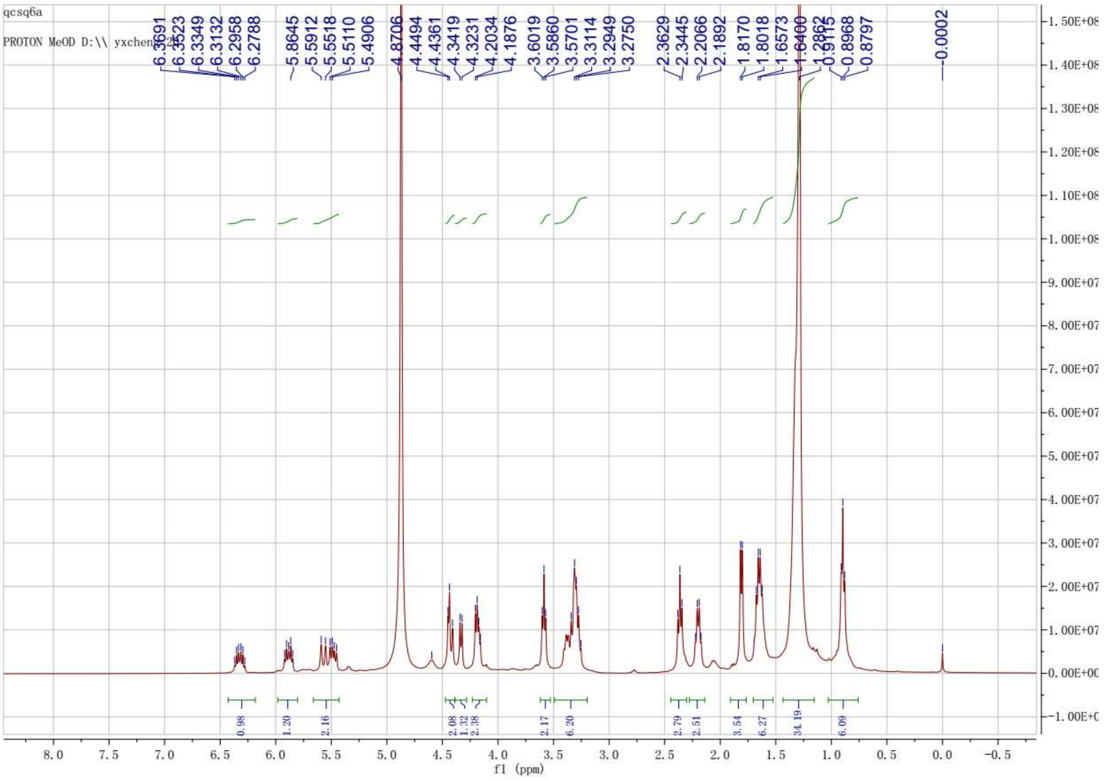

Figure S21. $^{1}$H NMR spectrum of **2** in Methanol-$d_4$

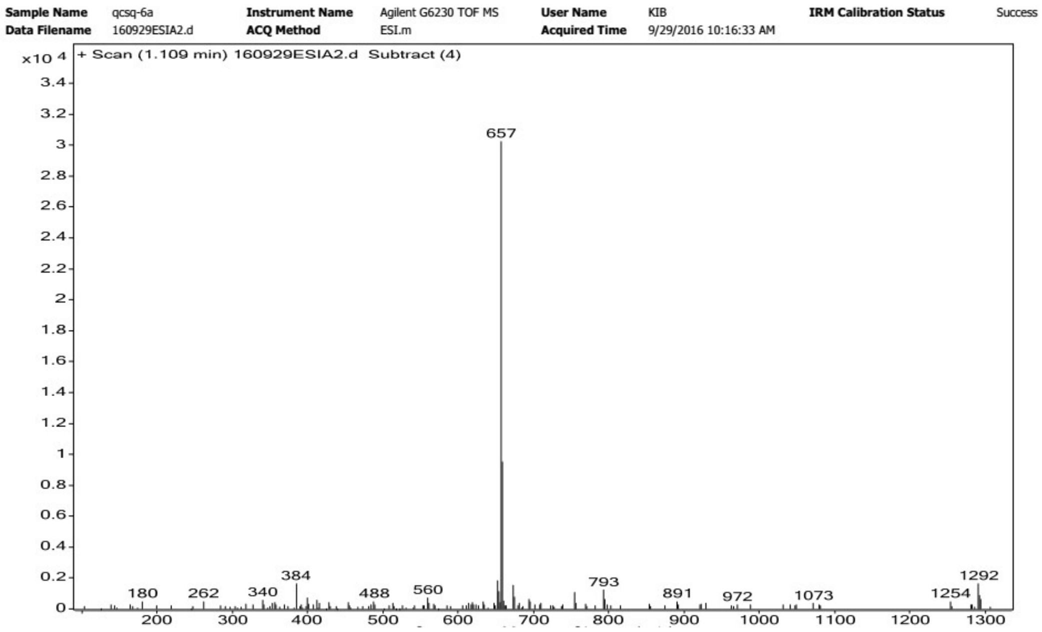


Figure S22. ESIMS of **2**

| | | | |
|---|---|---|---|
| **Data Filename** | 160929ESIA2.d | **Sample Name** | qcsq-6a |
| **Sample Type** | Sample | **Position** | |
| **Instrument Name** | Agilent G6230 TOF MS | **User Name** | KIB |
| **Acq Method** | ESI.m | **Acquired Time** | 9/29/2016 10:16:33 AM |
| **IRM Calibration Status** | Success | **DA Method** | ESI.m |
| **Comment** | | | |

**Sample Group** **Info.**

**Acquisition SW Version** 6200 series TOF/6500 series Q-TOF B.05.01 (B5125.2)

## User Spectra

| Fragmentor Voltage | Collision Energy | Ionization Mode |
|---|---|---|
| 200 | 0 | ESI |

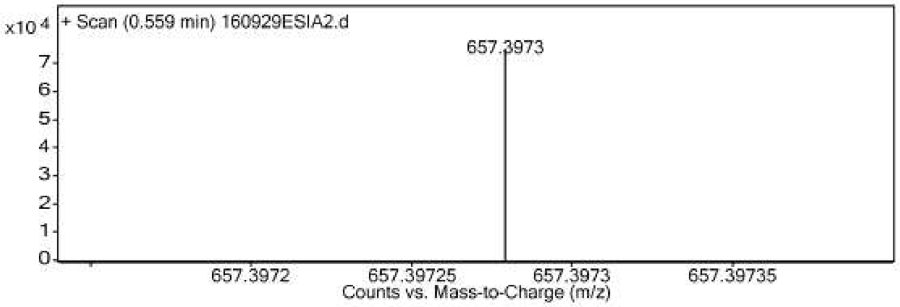


**Peak List**

| *m/z* | z | Abund | Formula | Ion |
|---|---|---|---|---|
| 105.0429 | | 5809.14 | | |
| 121.0509 | 1 | 68802.3 | | |
| 274.2736 | 1 | 11898.93 | | |
| 318.2999 | 1 | 9733.59 | | |
| 657.3973 | 1 | 74909.94 | C36 H58 Na O9 | M+ |
| 658.3999 | 1 | 22984.5 | C36 H58 Na O9 | M+ |
| 922.0098 | 1 | 71542.01 | | |
| 923.0106 | 1 | 9845.04 | | |
| 1291.8023 | 1 | 12512.37 | | |
| 1292.8052 | 1 | 9070.89 | | |

**Formula Calculator Element Limits**

| Element | Min | Max |
|---|---|---|
| C | 0 | 200 |
| H | 0 | 400 |
| O | 5 | 12 |
| Na | 1 | 1 |

**Formula Calculator Results**

| Formula | CalculatedMass | Mz | Diff.(mDa) | Diff. (ppm) | DBE |
|---|---|---|---|---|---|
| C36 H58 Na O9 | 657.3979 | 657.3973 | 0.5 | 0.8 | 7.5 |

--- End Of Report ---

Figure S23. HRESIMS of **2**

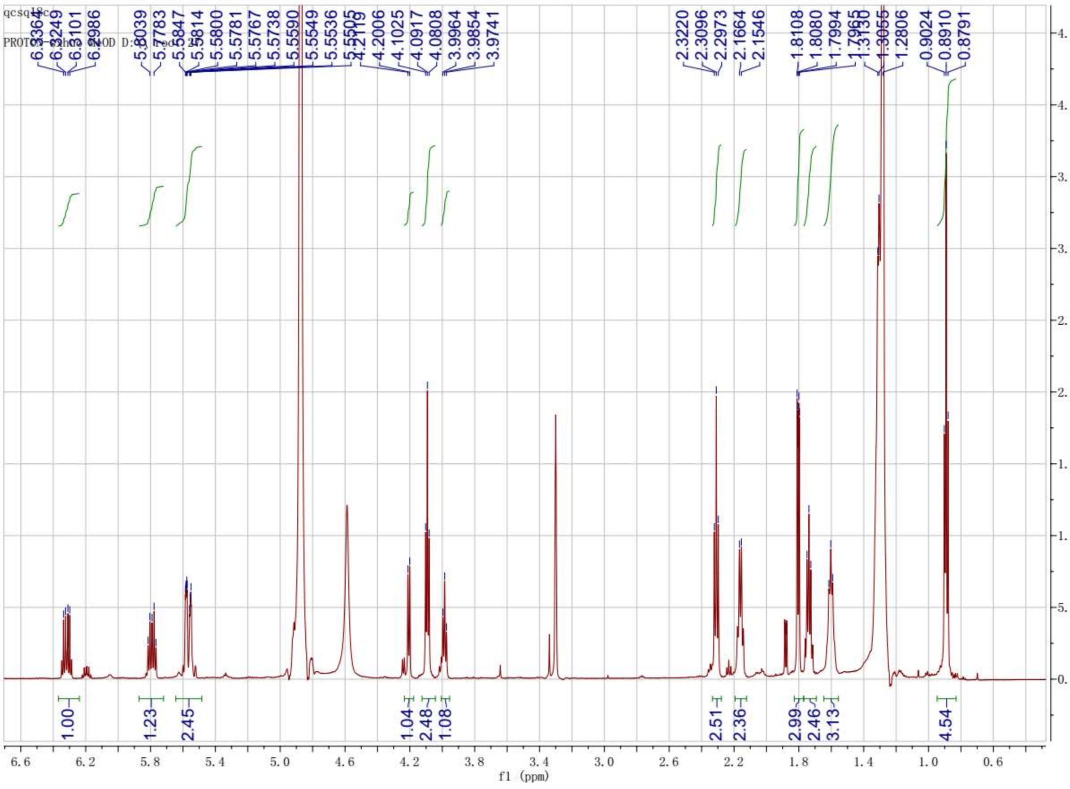


Figure S24.$^{1}$H NMR spectrum of **3** in Methanol-$d_4$

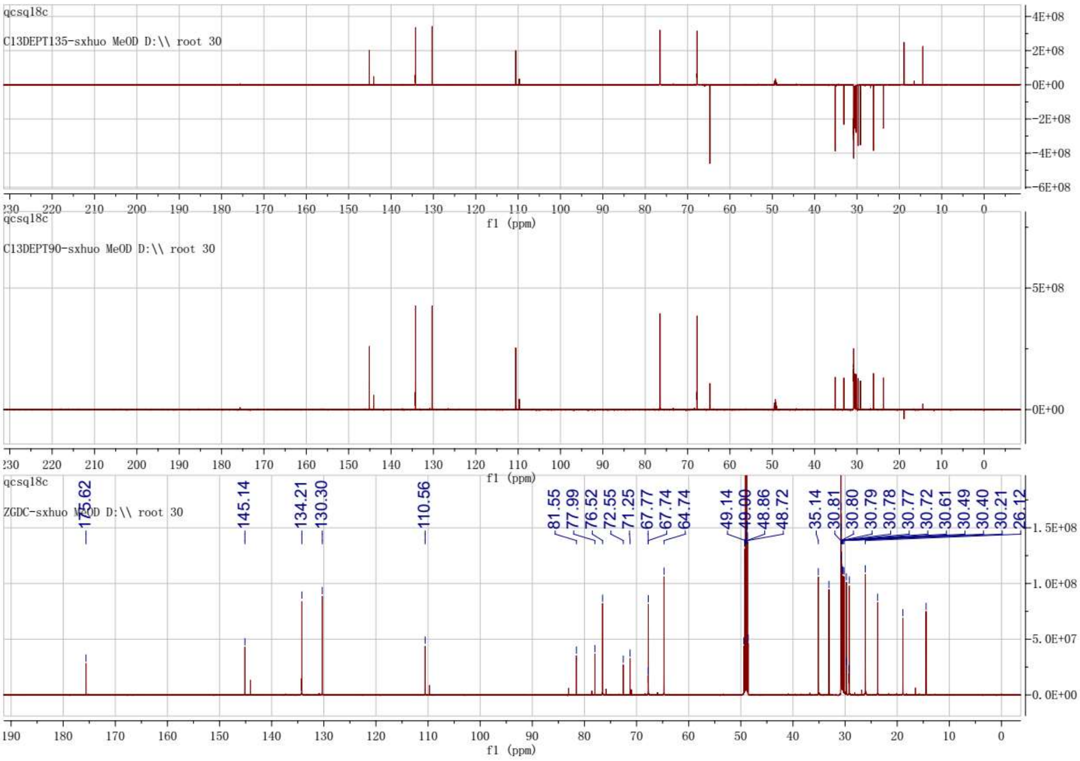


Figure S25. $^{13}$C NMR and DEPT spectra **3** in Methanol-$d_4$

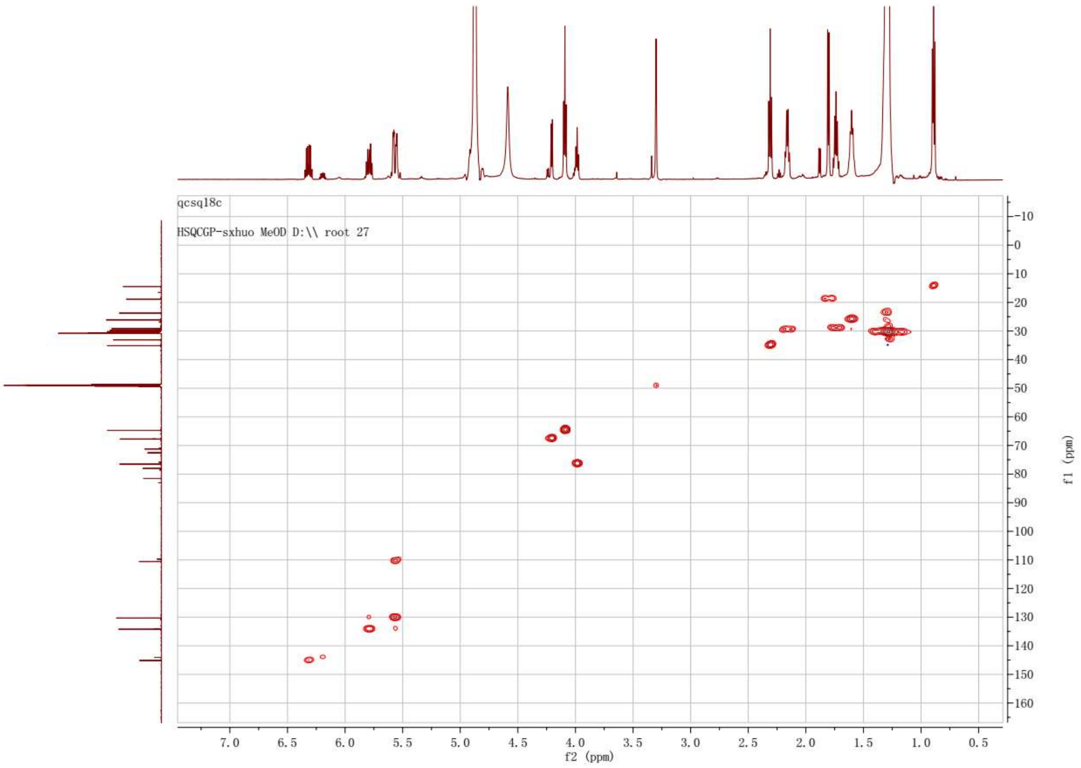


Figure S26. HSQC spectrum of **3** in Methanol-$d_4$

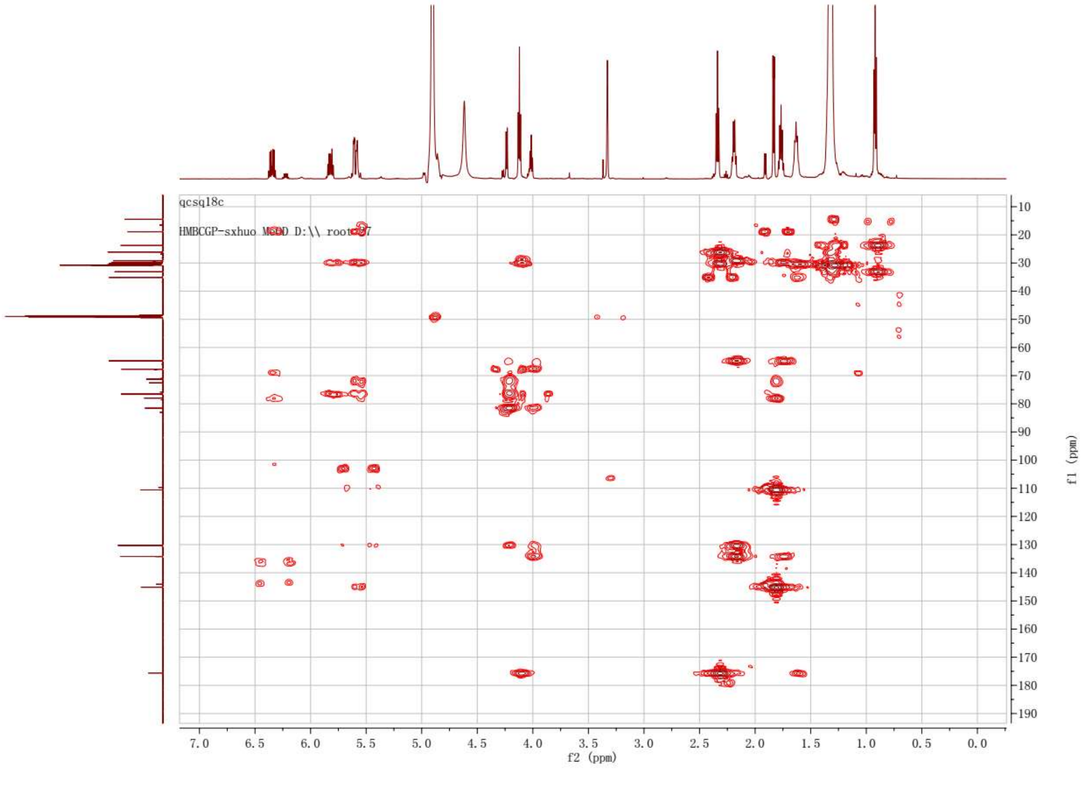


Figure S27. HMBC spectrum of **3** in Methanol-$d_4$

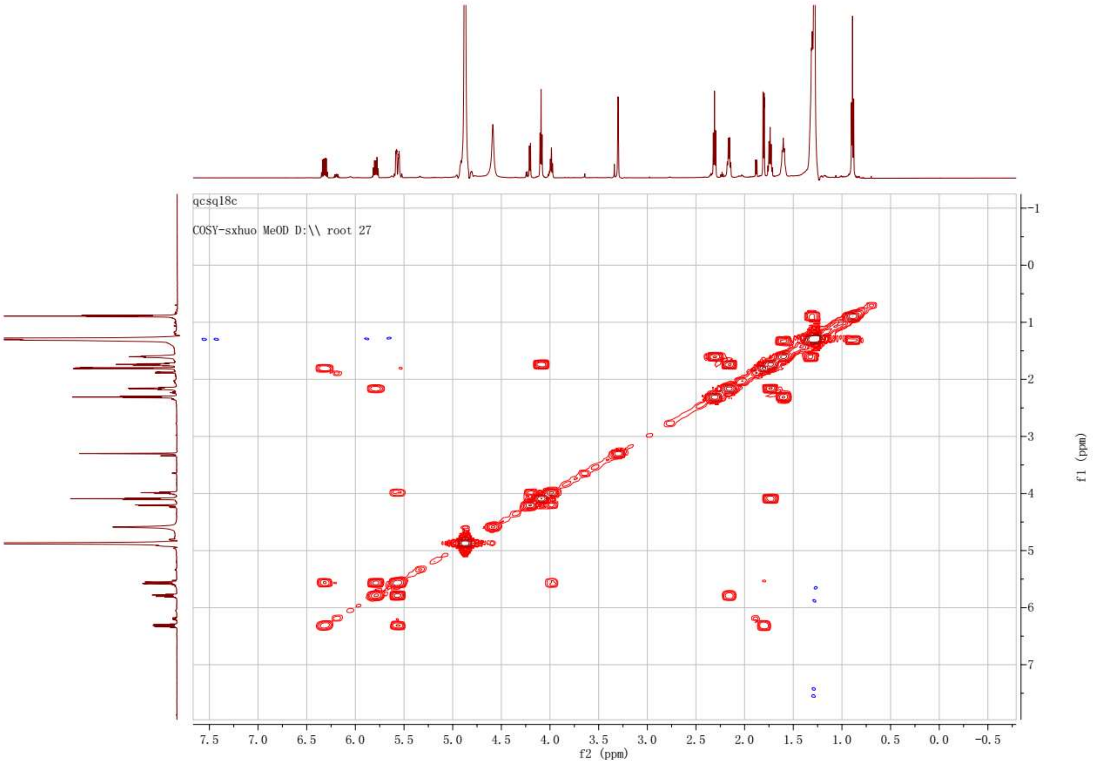


Figure S28. $^{1}$H-$^{1}$H COSY spectrum of **3** in Methanol-$d_4$

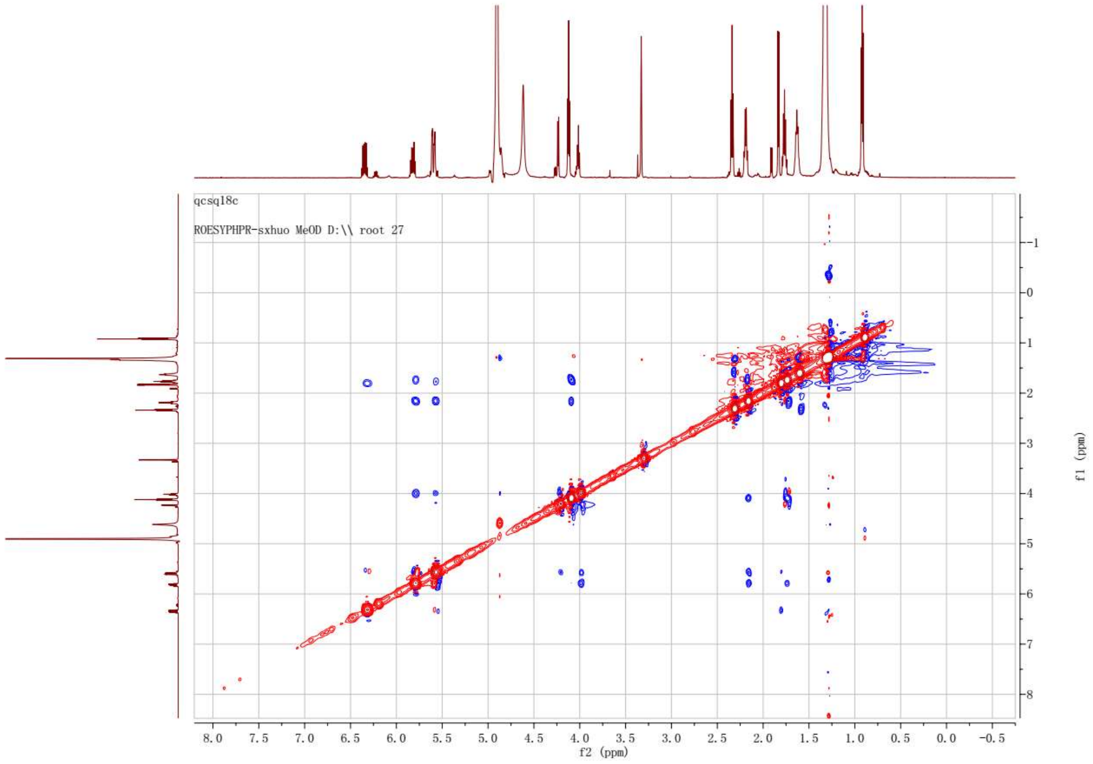


Figure S29. ROESY spectrum of **3** in Methanol-$d_4$

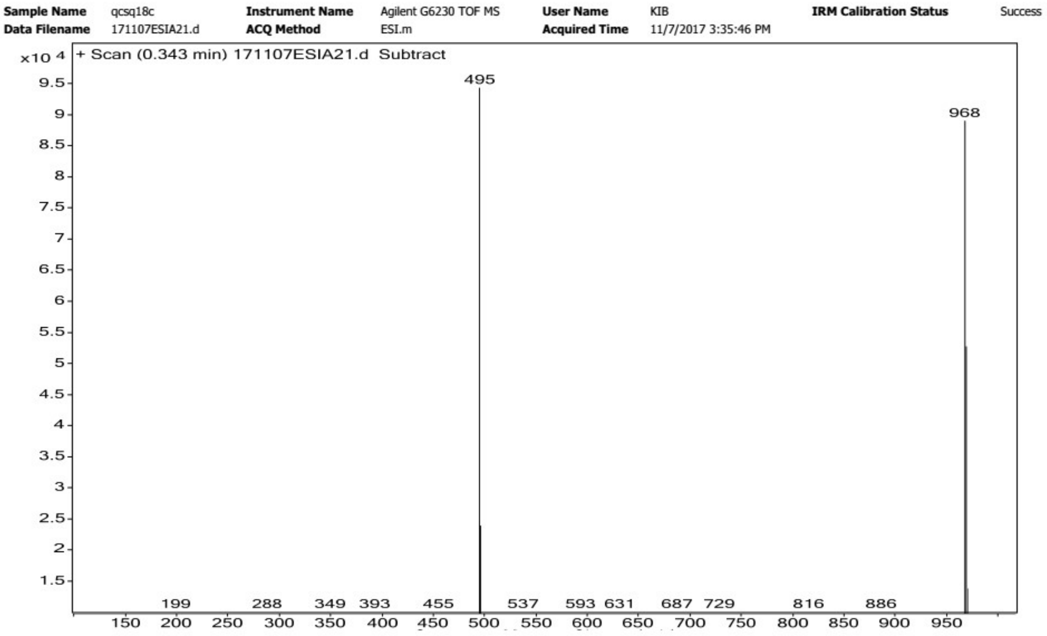


Figure S30. ESIMS of **3**

# Qualitative Analysis Report

| | | | |
|---|---|---|---|
| **Data Filename** | 171107ESIA21.d | **Sample Name** | qcsq18c |
| **Sample Type** | Sample | **Position** | |
| **Instrument Name** | Agilent G6230 TOF MS | **User Name** | KIB |
| **Acq Method** | ESI.m | **Acquired Time** | 11/7/2017 3:35:46 PM |
| **IRM Calibration Status** | Success | **DA Method** | ESI.m |
| **Comment** | | | |

**Sample Group** **Info.**

**Acquisition SW Version** 6200 series TOF/6500 series Q-TOF B.05.01 (B5125.2)

## User Spectra

| Fragmentor Voltage | Collision Energy | Ionization Mode |
|---|---|---|
| 200 | 0 | ESI |

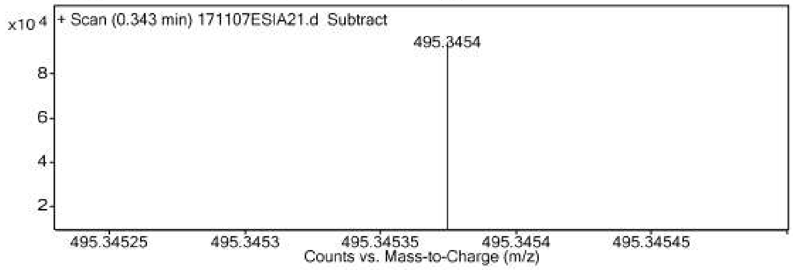


**Peak List**

| *m/z* | z | Abund | Formula | Ion |
|---|---|---|---|---|
| 490.3881 | 1 | 2102.16 | | |
| 495.3454 | 1 | 94289.97 | C30 H48 Na O4 | M+ |
| 496.3482 | 1 | 23804.53 | C30 H48 Na O4 | M+ |
| 497.3512 | 1 | 3631.07 | C30 H48 Na O4 | M+ |
| 511.3185 | 1 | 3265.76 | | |
| 967.702 | 1 | 88980.99 | | |
| 968.7046 | 1 | 52654.56 | | |
| 969.7065 | 1 | 13719.56 | | |
| 970.7078 | 1 | 2324.96 | | |
| 983.675 | 1 | 2921 | | |

**Formula Calculator Element Limits**

| Element | Min | Max |
|---|---|---|
| C | 0 | 200 |
| H | 0 | 400 |
| O | 0 | 10 |
| Na | 1 | 1 |

**Formula Calculator Results**

| Formula | CalculatedMass | Mz | Diff.(mDa) | Diff. (ppm) | DBE |
|---|---|---|---|---|---|
| C30 H48 Na O4 | 495.3450 | 495.3454 | -0.4 | 0.7 | 6.5 |

--- End Of Report ---

Figure S31. HRESIMS of **3**
